# Suppression of ATG4B by copper inhibits autophagy and involves in Mallory body formation

**DOI:** 10.1016/j.redox.2022.102284

**Published:** 2022-03-24

**Authors:** Fan Xia, Yuanyuan Fu, Huazhong Xie, Yuxin Chen, Dongmei Fang, Wei Zhang, Peiqing Liu, Min Li

**Affiliations:** a School of Pharmaceutical Sciences, Guangdong Provincial Key Laboratory of Chiral Molecule and Drug Discovery, National and Local Joint Engineering Laboratory of Druggability and New Drugs Evaluation, Guangdong Engineering Laboratory of Druggability and New Drugs Evaluation, Sun Yat-Sen University, Guangzhou, 510006, China; b Laboratory Animal Center, Sun Yat-Sen University, Guangzhou, 510006, China

**Keywords:** Aggregates, ATG4B, Autophagy, Copper ion, Mallory body

## Abstract

Autophagy is an evolutionarily conserved self-protecting mechanism implicated in cellular homeostasis. ATG4B plays a vital role in autophagy process via undertaking priming and delipidation of LC3. Chemical inhibitors and regulative modifications such as oxidation of ATG4B have been demonstrated to modulate autophagy function. Whether and how ATG4B could be regulated by metal ions is largely unknown. Copper is an essential trace metal served as static co-factors in redox reactions in physiology process. Excessive accumulation of copper in *ATP7B* mutant cells leads to pathology progression such as insoluble Mallory body (MB) in Wilson disease (WD). The clearance of MB via autophagy pathway was thought as a promising strategy for WD. Here, we discovered that copper ion instead of other ions could inhibit the activity of ATG4B followed by autophagy suppression. In addition, copper could induce ATG4B oligomers depending on cysteine oxidation which could be abolished in reduced condition. Copper also promotes the formation of insoluble ATG4B aggregates, as well as p62-and ubiquitin-positive aggregates, which is consistent with the components of MB caused by copper overload in WD cell model. Importantly, overexpression of ATG4B could partially reduce the formation of MB and rescue impaired autophagy. Taken together, our results uncovered for the first time a new damage mechanism mediated by copper and implied new insights of the crosstalk between the toxicity of copper and autophagy in the pathogenesis of WD.

## Highlights

• Copper inhibits the activity of ATG4B *in vitro* and in cells.
• Copper blocks autophagy via the inhibition of ATG4B.
• Copper-induced ATG4B aggregation and oligomerization depend on cysteine oxidation.
• Overexpression of ATG4B could reduce the formation of Mallory body in copper overload Wilson disease model.

## Abbreviations:

ATG: autophagy-related
ATP7B: ATPase copper-transporting beta
CBB: coomassie brilliant blue
CQ: chloroquine
Ctr1: copper transport receptor 1
Cu: copper
DTT: dithiothreitol
FRET: fluorescence resonance energy transfer
LC3: microtubule-associated protein 1 light chain 3
LLPD: long-lived protein degradation
Mallory body: MB
NAC: N-acetylcysteine
PE: phosphatidylethanolamine
PLD: phospholipase D
PTMs: posttranslational modifications
SDS-PAGE: sodium dodecyl sulfate polyacrylamide gel electrophoresis
TX: Triton X-100
WD: Wilson disease
WT: wild-type

## 1 Introduction

Macroautophagy (hereafter referred to as autophagy) is a self-protecting mechanism that degrades cytosolic components enclosed by a de novo double-membrane vesicle termed as autophagosome in a lysosome-dependent manner [1]. Until now, 41 autophagy-related (*ATG*) genes that encode proteins employed in diverse functions in autophagy named *ATG1* to *ATG42* have been identified from yeast genetic screens [2]. Accordingly, the abbreviations would be “*ATG*” and “ATG” for gene and protein when referring to humans, “*ATG*” and “Atg” for yeast, and “*Atg*” and “ATG” for the mouse system [3]. Among them, a troop of structurally and functionally reserved proteins participating in autophagosome formation are classically termed as core autophagy molecular machinery, which are categorized into five functional units: Atg1 kinase and its regulators, the Atg2-Atg18 complex, the PI3K complex, the Atg8 and Atg12 ubiquitin systems and Atg9 [4]. In mammals, the cysteine protease ATG4B plays dual roles to each of the seven ATG8 orthologues in mammalian, respectively MAP1LC3A/LC3A (microtubule-associated protein 1 light chain 3A), LC3B, LC3B2, LC3C, GABRAP, GABRRAPL1 and GABARAPL2/GATE-16 [5]. ATG4B cleaves off pro-LC3/GABARAP at C-terminal residues to produce glycine-exposed active form of LC3-I/GABARAP-I, enabling the attachment of LC3/GABARAP to phosphatidylethanolamine (PE) on both sides of the phagophore membrane to form LC3/GABARAP-II. ATG4 also shears LC3/GABARAP –II from the outer autophagosome membrane, named delipidation, to recycle LC3/GABARAP –I [6]. Mammals have four ATG4 homologs, namely ATG4A, ATG4B, ATG4C and ATG4D, of which ATG4B possesses the highest efficiency and broadest specificity [7]. The delipidation of LC3/GABARAP mediated by ATG4 is of great significance in autophagic processes, including isolation membrane expansion, the accurate localization of yeast Atg8 to the vacuolar membrane, autophagosome biogenesis and maturation, and the assembly of Atg9-containing tubulovesicular clusters into autophagosome [8,9]. Therefore, inhibition of ATG4B-mediated LC3/GABARAP processing would cause autophagy defects.

Studies have revealed that the enzymatic activity and protein level of ATG4B could be adjusted by various factors. Small molecules, such as NSC185058 and S130, have been identified to inhibit ATG4B and suppress autophagy [10,11]. The function of ATG4B is also regulated by multiple posttranslational modifications (PTMs), including oxidation [12,13], O-GlcNAcylation [14], S-nitrosation [15], phosphorylation [16], and ubiquitination [17]. Besides, Atg4 has been discovered to form intramolecular disulfide bonds in *Saccharomyces cerevisiae*, *Chlamydomonas reinhardtii*, and *Arabidopsis* upon oxidation [[18], [19], [20]]. Furthermore, alga Atg4 and human ATG4B could aggregate and form oligomers under oxidative stress which further affect autophagy signals [13,20].

Among the structurally identified proteins in PDB database, one in three contains a metal served as a cofactor [21], including enzymes (e.g., proteins carrying Fe, Cu, and Zn) and transcriptional factors (e.g., proteins containing Zn). Their ability of existing in multiple oxidation states and diverse geometries enable them to participate in redox reactions and biological processes [22]. Since most transition metals have unfilled d-orbitals and thus are redox active in cells. We wondered whether ATG4B activity could be regulated by the oxidation of these transition metals and thereby leading to autophagy-related physiological or pathological changes.

Copper (Cu) is an essential trace metal for human physiology that is served as co-factors in the catalysis of several critical cellular enzymes, such as cytochrome C oxidase, peptidyl-a-mono oxygenase, tyrosinase, and dopamine-β-monooxygenase. The reason that copper could take part as a co-factor in redox reactions attributing to its special electron structure that allows the interplay with spin-restricted molecular oxygen [23]. However, this chemical nature makes copper toxic as well. There are several diseases whose pathology are directly associated with copper overload， such as Wilson disease (WD), Indian childhood cirrhosis, endemic Tyrolean infantile cirrhosis, and idiopathic copper toxicosis [24]. The etiology of WD is mutations in copper-transporting ATPase beta (*ATP7B*) while the genetic defects of other three diseases remain unclear [25]. The toxicity of copper overload is in part a consequence of the release of reactive oxygen species via Fenton or Haber-Weiss reactions leading to lipid, protein, DNA, and RNA damage [26]. An alternative mechanism of copper toxicity is apoptosis induced by activation of acid sphingomyelinase and mitochondrial damage resulting in frustrated hepatic energy metabolism and cholesterol biosynthesis [27]. Meanwhile, the nonspecifically direct binding of copper to the thiol and amino groups of proteins, thereby destroying their structures and biology functions [23]. The pathology developments of copper overload diseases in different stages are closely related to copper exposure, namely from inflammation and steatosis to fibrosis, cirrhosis and Mallory body (MB) formation [28]. MBs are cellular hyaline inclusions of hepatocytes which are primarily observed in alcoholic steatohepatitis and are now widely found in other hepatic diseases including nonalcoholic steatohepatitis, primary biliary cirrhosis, hepatocellular carcinoma, and WD [29]. Ubiquitin-binding protein p62, ubiquitin, and intermediate filament proteins keratin 8 and 18 (K8/18) are integral components that make up MBs [30]. The morphology, ultrastructure, and biochemical characterization of MBs observed in Indian childhood cirrhosis, idiopathic copper toxicosis, and WD have been well studied [31]. However, the specific mechanisms of the formation and clearance of MBs in copper intoxication are poorly understood. It has been reported that autophagy participates in the elimination and resorption of 3, 5-diethoxycarbonyl-1, 4-dihydrocollidine and proteasome inhibitors-triggered MBs [32,33]. In WD, it has been discovered that activation of autophagy protects *ATP7B* knockout cells from copper-induced apoptosis [34]. Copper directly interacts with autophagic kinase ULK1/2 to activate autophagy [35]. Therefore, we wondered whether copper-induced autophagy activation plays a role in MB formation in WD.

In this study, we firstly found copper strongly inhibits the activity of ATG4B resulting in LC3-II accumulation and autophagy suppression. Copper could also trigger the formation of soluble ATG4B oligomers, and insoluble aggregates like the MBs in WD cell models. Interestingly, overexpression of ATG4B could partially reduce the accumulation of MB via reversing impaired autophagy in *ATP7B* knockdown cell model of WD. Our work uncovers a new signaling pathway in copper overload diseases, namely copper inhibits ATG4B and subsequently suppresses autophagy. This process not only allows us to comprehensively understand the pathological mechanism of copper overload and autophagy, but provides a novel therapeutic strategy for WD.

## 2 Results

### 2.1 Copper effectively inhibits the activity of ATG4B

**Fig. 1:**
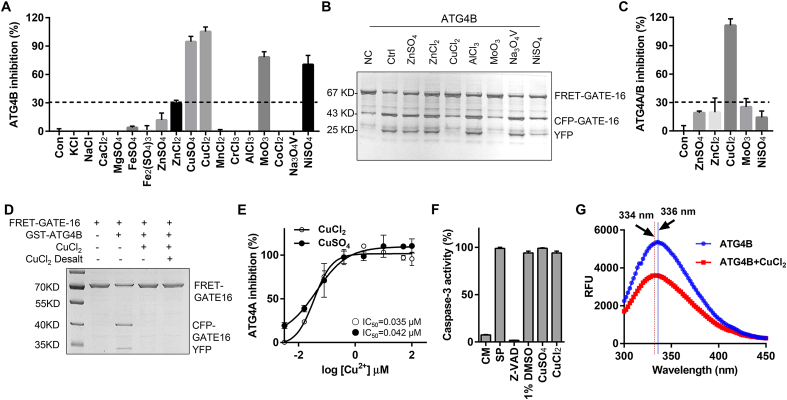
**Copper effectively inhibits the activity of ATG4B.** (**A-B**) ATG4B (0.5 μg/ml) was incubated with different metal ions (10 μM) for 30 min in a total volume of 50 μl at 37 °C followed by adding FRET-GATE-16 (50 μg/ml). RFUs at the excitation wavelength of 477 nm and the emission from 527 nm using a fluorescence spectrometer (A) or to stop the reaction via adding loading buffer. Then protein samples were subject to CBB analysis (B). NC means negative control without enzyme. (**C**) Cell lysates, extracted from HeLa cells with treatment of different metal ions (20 μM) for 6 h, were incubated with FRET-GATE-16 (50 μg/ml) for 30 min at 37 °C. The RFUs at 527 nm and 477 nm were recorded and the ratios were calculated. (**D**) Glutathione beads binding with GST-ATG4B were treated by copper ions followed by PBS buffer wash without copper ion. Then GST-ATG4B was eluted for activity detection. The activity of ATG4B was measured with or without copper desalting. (**E**) The IC_50_ of CuCl_2_ or CuSO_4_ to ATG4B from fitted curve was calculated by FRET. (**F**) Analysis of the protease activity of cysteine protease Caspase-3 before and after 1 μM of copper treatment. (**G**) Recombination protein ATG4B (1 mg/ml) was incubated with or without copper ion (1 μM) at room temperature in a volume of 100 μl for 30 min. Data were collected at the excitation wavelength of 280 nm and the emission from 300 nm to 450 nm using a fluorescence spectrometer.

To screen ATG4B inhibitors, we performed a fluorescence resonance energy transfer (FRET) assay [36], which is based on substrate protein FRET-GATE-16 fused with CFP and YFP cleaved by ATG4A and ATG4B. We firstly found that CuCl_2_, CuSO_4_, MoO_3_, and NiSO_4_ could exclusively inhibit the activity of ATG4A and ATG4B among diverse metal ions *in vitro* (Figs. 1A and S1A). We could intuitively observe CuCl_2_ and MoO_3_ suppress the enzyme activity of ATG4A and ATG4B via Coomassie brilliant blue (CBB) assay (Figs. 1B and S1B). Then, we measured the activity of endogenous ATG4A and ATG4B using HeLa cell lysates after various metal ions exposure, and found the activity only decreased under copper treatment (Fig. 1C). To rule out the possibility that the substrates FRET-GATE-16 might be inhibited by copper in the *in vitro* cleavage assay, glutathione beads binding with GST-ATG4B were treated by copper followed by PBS buffer wash without copper ion. Then GST-ATG4B was eluted for activity detection. As shown in Fig. 1D, the activity of ATG4B could be also suppressed significantly after copper desalting, suggesting that copper ions can tightly bind to ATG4B.

In addition, to figure out whether copper could inhibit other proteases, we detected the enzyme activity of several cysteine proteases such as caspase-3, -2, -8, -9, and serine proteases such as plasmin, kallikrein, and factor Xa before and after copper treatment and found almost no *in vitro* inhibition was found (Figs. 1F and S1D). Although copper could also inhibit ATG4A (Figs. S1A–B), the IC_50_ of copper to ATG4B is almost 10-fold less than that to ATG4A (Figs. 1E and S1C). To detect the binding pattern between copper and ATG4B, we carried out surface plasmon resonance assay using recombinant protein ATG4B with or without His-tag (His-ATG4B and ATG4B) and ATG4B mutant His-ATG4B^C74S^, respectively. Copper exhibited a robust and irreversible binding affinity for ATG4B and His-ATG4B that copper failed to dissociated from ATG4B (Figs. S1E–F). In addition, we found that single-site mutant (ATG4B^C74S^) did not significantly affect the binding of copper to ATG4B (Fig. S1G). Meanwhile, copper cannot be dissociated from all three ATG4B proteins, suggesting copper could irreversibly bind to ATG4B independent of Cys74 and histidine. Furthermore, we also investigated the conformation change of ATG4B based on fluorescent spectra and found that a 2 nm shift occurred under copper treatment, implying Cu might change the conformation of ATG4B (Fig. 1G). As we know, ATG4B is the prominent ATG4 homolog with the highest efficiency and broadest specificity [7]. Therefore, as the target of copper, ATG4B was chosen to further investigate its novel physiological and pathological function.

These results indicated that copper instead of other metal ions could strongly interact with ATG4B and inhibit its enzymatic activity.

### 2.2 Copper represses autophagy flux via inhibiting ATG4B

**Fig. 2:**
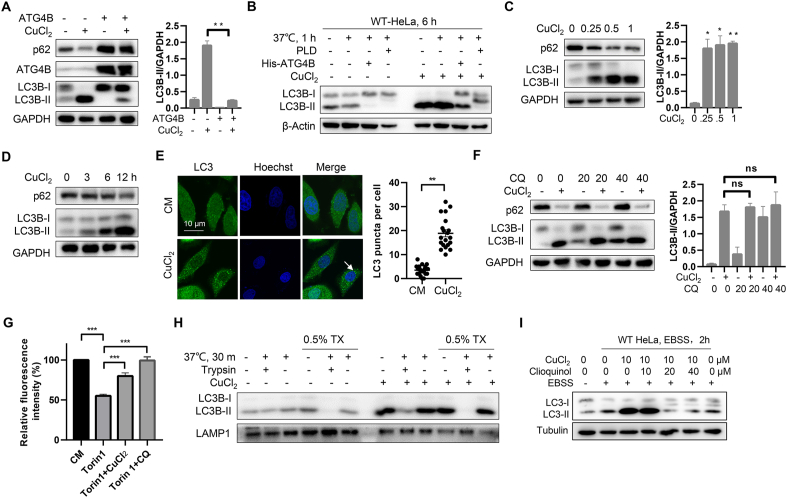
**Copper represses autophagy flux via inhibiting ATG4B.** (**A**) Western blotting of cell lysates from WT HeLa cells and ATG4B-Flag HeLa cells treated with or without copper ions (1 mM) for 6 h. (**B**) PLD band shift assay performed on WT HeLa cells and *ATG4B* KO HeLa cells treated with copper ions (1 mM) for 6 h and lysed in PLD assay buffer. Lysates were subject to *in vitro* treatment with PLD or His-ATG4B prior to western blotting. (**C**) HeLa cells treated with indicated concentrations of copper ions for 6 h were analyzed by immunoblot. (**D**) HeLa cells treated with 500 μM of copper ions for 0–12 h were assessed by immunoblot. (**E**) The formation of endogenous LC3B puncta in HeLa cells treated with copper ions (1 mM) for 6 h was revealed by immunostaining. Scale bar: 10 μm. Quantification of LC3B puncta per cell shown on right hand side. n = 50 randomly-selected cells per condition. (**F**) Western blotting of lysates from HeLa cells treated with copper ions (1 mM) for 6 h in the presence or absence of 20 or 40 μM of CQ. LC3-II/GAPDH was calculated based on the band density. (**G**) Hela cells were treated by 4 μM of Torin 1 in the presence or absence of 1 mM of copper ions and 20 mM of CQ for 12 h, then the degradation ratio of long-lived proteins was measured using flow cytometry. (**H**) Protease protection assay of homogenates from HeLa cells treated with copper ions (1 mM) for 6 h. (**I**) WT HeLa cells were treated in EBSS with or without 10 μM of copper and 0–40 μM of clioquinol. Protein level of LC3-II was recorded. ***P < 0.001. **P < 0.01, *P < 0.05, n.s. (not significant) (*t*-test).

In order to investigate the overall effect of copper on autophagy process, we treated wild-type (WT) HeLa and HeLa cells stably expressed ATG4B-Flag with CuCl_2_ to assess the alteration of the autophagy marker LC3. As the lipidated form of LC3 paralogues were sequestered by excess ATG4B to form ATG4B-LC3-I complex and therefore the formation of LC3-II was blocked in ATG4B overexpressed cells [37]. We observed that the lipidated form of LC3-II was rescued under copper treatment in ATG4B-Flag HeLa cells (Fig. 2A), suggesting that copper could disrupt ATG4B-LC3-I complex in cells. In addition, the deconjugation of lipidated LC3 (LC3-II) to LC3-I might be strongly enhanced due to the higher concentration of ATG4B. As shown in Fig. 2A, treatment of HeLa cells with CuCl_2_ results in a higher level of pro-LC3 or LC3-II due to the similar migration rate of human pro-LC3B and LC3B-II in SDS-PAGE. ATG4B could execute two LC3 proteolytic processing events: priming of pro-LC3 to LC3-I and deconjugation of LC3-II to LC3-I. To differentiate whether the lower bands of LC3 were pro-LC3 or LC3-II, cell lysates were mixed with ATG4B or phospholipase D (PLD) followed by Western blot analysis. This assay is based on the fact that ATG4B cleaves both pro-LC3 and LC3-II while PLD only shear lipidated LC3-II [5,38]. The dominant LC3 bands in *ATG4B*KO HeLa cells (pro-LC3) were thoroughly resistant to PLD treatment but could be cleaved under ATG4B treatment. On the contrary, LC3 bands are sensitive to both ATG4B and PLD in WT HeLa cells (Figs. 2B and S2A) indicating that copper could accumulate the formation of lipidated LC3-II rather than pro-LC3 by inhibiting ATG4B-mediated delipidation. The above processes were illustrated in Fig. S2B. Additionally, immunoblot analysis of copper-treated cells revealed that copper could promote the lipidation of LC3 and the degradation of autophagy cargo receptor p62/SQSTM1 in a time- and dosage-dependent manner in HeLa cells (Fig. 2C–D). An immunofluorescence analysis of endogenous LC3 puncta uncovered a consistent induction of LC3 dots formation with copper treatment (Fig. 2E). In respect to LC3 lipidation, we could not observe an accumulation of lower band of LC3 in response to copper exposure in *ATG16L1*KO HeLa (Fig. S2C), confirming that copper-induced LC3 lipidation is autophagy dependent. Meanwhile, copper increases the formation of ULK1 puncta (Fig. S2D), which is consistent with the previous finding that copper could activate the upstream signals of autophagy ULK1 [35].

We then treated HeLa cells with or without chloroquine (CQ), an autophagy inhibitor by increasing the lysosome pH and impairing the degradative activity of lysosome, in the presence of copper and found that the level of LC3B-II did not further increase after copper treatment (Fig. 2F). Such autophagy flux assay was also confirmed by L02 cells (Fig. S2E), suggesting autophagy was indeed suppressed by copper. To further accurately dissect the autophagy activity, the long-lived protein degradation (LLPD) assay was performed. As shown in Fig. 2G, Torin 1 could enhance the degradation of long-lived proteins as expected, while such activity was strongly suppressed by copper and CQ, indicating that copper did not induce autophagy but blocked autophagy. Since ATG4B-regulated deconjugation has previously been involved in autophagosome closure [9], we reasoned that copper-mediated inactivation of ATG4B resulted in LC3-II accumulation and thereby prevented autophagosome from sealing. In protease protection assay which was designed to differentiate proteins between the outside and the inside of closed double-membrane vesicles. LAMP1 was used as a loading control since it was resistant to TX-100. We observed a fraction of LC3-II induced by copper was resistant to trypsin in the absence of detergent (Fig. 2H), suggesting the closure of autophagosome was severely but not completely impaired under copper treatment in consistent with *ATG4B*KO HeLa cells since the distribution of LC3-II was imbalanced. Besides, a well-known copper chelator clioquinol was applied and found the impaired autophagy caused by copper was significantly reverted (Fig. 2I).

These results demonstrate that although copper could activate the upstream signals of autophagy such as ULK1, copper also inhibits the late-stage of autophagy flux via suppressing ATG4B-mediated LC3-II delipidation.

### 2.3 Copper promotes the formation of p62 and ATG4B aggregates

**Fig. 3:**
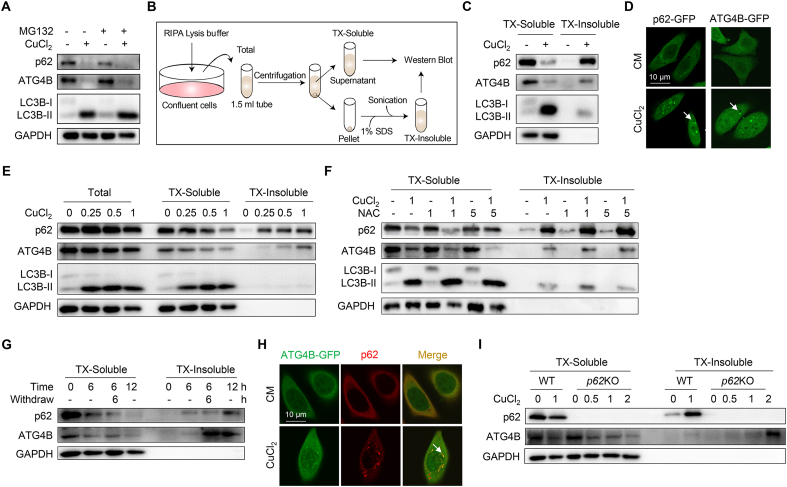
**Copper leads to the formation of p62 and ATG4B aggregates.** (**A**)Western blotting of lysates from HeLa cells treated with 1 mM of copper ions with or without 10 μM of MG132 for 6 h. (**B**) Diagram showing the procedure of differential detergent fractionation. (**C**) Differential detergent fraction followed by western blotting was performed on HeLa cells treated for copper ions for 6 h. (**D**) Image of HeLa cells stably expressing GFP-tagged p62 or ATG4B before and after treatment of 1 mM copper ions for 6 h. (**E**) Differential detergent fractionation was performed on HeLa cells treated with designed concentrations of copper ions for 6 h and western blotting was carried out to detect the protein levels. (**F**) Differential detergent fractionation was performed on HeLa cells treated with 1 mM of copper ions with or without designed concentrations of NAC for 6 h and western blotting was carried out to detect the protein levels. (**G**) HeLa cells were treated with 1 mM of copper ions for 6 h, 12 h, or 6 h followed by PBS washout for three times and then cultured for 6 h. Differential detergent fractionation was performed on treated cells and western blotting was carried out to detect the protein levels. (**H**) Colocalization of endogenous p62 in GFP-ATG4B HeLa cells after treatment of 1 mM of copper ions for 6 h. (**I**) Differential detergent fractionation was performed on WT L02 cells and p62 knockout L02 cells treated with designed concentrations of copper ions for 6 h and western blotting was carried out to detect the protein levels.

We previously demonstrated that autophagy flux is impaired in response to copper. However, we found p62, another frequently-used maker to characterize autophagy activity, was abnormally degraded under copper treatment even in the presence of autophagy inhibitor CQ (Figs. 2F and S2C). We then employed proteasome inhibitor MG132 to dissect whether p62 was degraded through proteasome. As shown in Fig. 3A and S3A, the rapid breakdown of p62 and ATG4B was unable to be reversed by MG132, suggesting copper-mediated degradation of p62 and ATG4B is independent of the proteasome pathway.

P62 is a common component of inclusion bodies under autophagy or proteasome deficiency or in protein aggregation diseases, which are often characterized as insoluble in mild detergents containing lysis buffer [39]. We performed a differential detergent fractionation approach to separately analyze the distribution of p62 and ATG4B in mild Triton X (TX)-100 lysis buffered supernatant (TX-Soluble) and harsher 1% SDS lysis buffered pellet (TX-Insoluble) (Fig. 3B). It turned out that p62 and ATG4B could be accumulated in TX-Insoluble fraction to form protein aggregates using differential detergent fractionation approach prior to immunoblot analysis (Fig. 3C). To directly observe the aggregates, we obtained HeLa cells stably expressed GFP-tagged p62. The formation of small protein aggregates under copper treatment was found (Fig. 3D). This demonstrates that copper promotes the accumulation of both p62-and ATG4B-positive protein aggregates. Next, the reduction in the supernatant and the accumulation in TX-Insoluble pellets of p62 and ATG4B occurred in a dosage- and time-dependent way (Figs. 3E and S3B). This tendency was also appeared in L02 and HepaRG cells (Figs. S3C–D).

Given the ability of copper to generate excessive amounts of reactive oxygen species and the fact that ATG4B could form oligomers under oxidative stress [13,24], we wondered whether the formation of ATG4B aggregation is due to copper-induced oxidative stress. We then treated HeLa cells with ROS scavenger N-acetylcysteine (NAC) in the absence and presence of copper and found that NAC was unable to block the formation of both p62 and ATG4B aggregates under copper treatment (Fig. 3F). In addition, ATG4B aggregates in inclusions following copper withdrawal could not be reduced, suggesting the aggregates caused by copper are irreversible (Fig. 3G).

P62 has a tendency to form cytoplasmic aggregation depending on its PB1 domain for self-oligomerization and UBA domain for binding to ubiquitinated proteins or polyubiquitin chains under various stimulation [40]. In addition, p62 has been reported to directly interact with ATG4B [41]. We reasoned whether copper-induced aggregation of ATG4B is due to the aggregation of p62 under copper-mediated autophagy deficiency, which subsequently promotes the formation of ATG4B aggregation via the interaction between ATG4B and p62. Immunofluorescence analysis showed that ATG4B was colocalized with p62 inclusions (Fig. 3H). However, there is still ATG4B aggregation in TX-Insoluble fraction after copper treatment in *p62*KO cells (Fig. 3I).

These results suggest that copper could promote the formation of p62 and ATG4B aggregates. Copper-mediated aggregation of ATG4B is not dependent on p62.

### 2.4 Copper-induced ATG4B aggregation depends on the peptidase_C54 domain of ATG4B

**Fig. 4:**
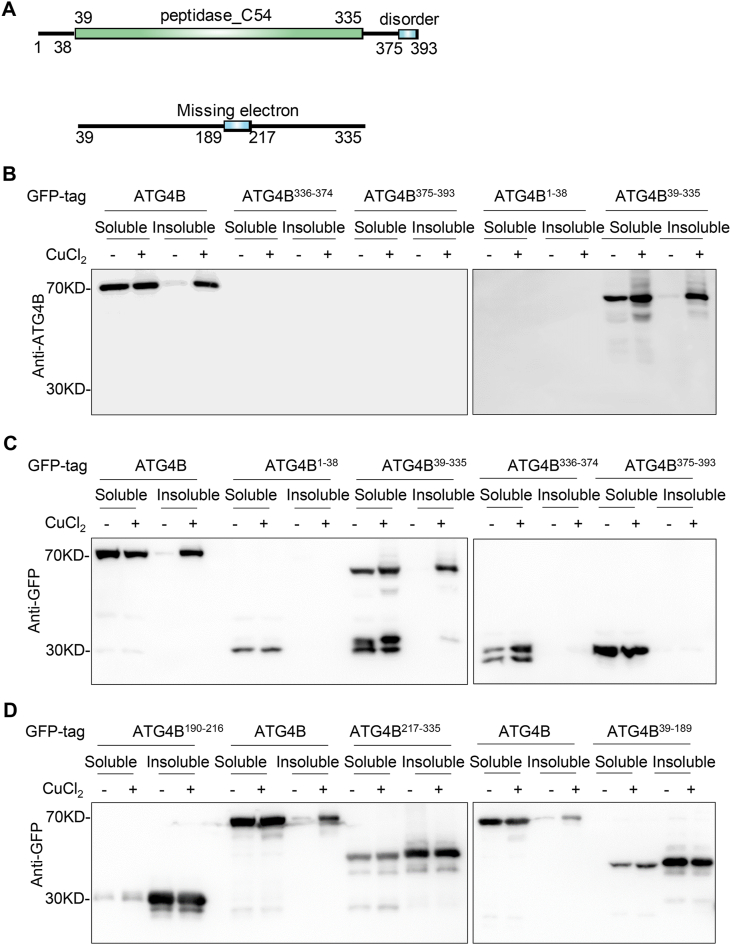
**Copper-induced ATG4B aggregation is dependent on peptidase_C54 domain of ATG4B.** (**A**) Schematic diagram of the truncation of ATG4B (a.a. 1–38, a.a. 39–335, a.a. 336–374, a.a. 375–393, a.a. 39–189, a.a. 190–217, a.a. 218–335). The numbers indicate amino acid residues of ATG4B. (**B-D**) Differential detergent fractionation was performed on *ATG4B*KO HeLa cells stably expressing four GFP-tagged truncations of ATG4B after treatment with 1 mM of copper ions for 6 h and western blotting was carried out to detect the protein levels of ATG4B (B) or GFP (C–D).

To identify the domain of ATG4B responsible for ATG4B aggregation, we constructed four ATG4B truncations on the basis of the C54 peptidase domain and C-terminal disorder region of ATG4B (Fig. 4A). GFP-fused ATG4B and its truncation ATG4B^1-38^, ATG4B^39-335^, ATG4B^336-374^, and ATG4B^375-393^ were expressed in *ATG4B*KO HeLa cells followed by copper treatments. Interestingly, only the C54 peptidase domain (ATG4B^39-335^) was aggregated in inclusions using ATG4B antibody (Fig. 4B), and confirmed by GFP antibody (Fig. 4C). We further subdivided the C54 peptidase domain of ATG4B into three GFP-tagged truncations, ATG4B^39-189^, ATG4B^189-216^, and ATG4B^217-335^, based on its middle missing electron region (Fig. 4A). However, all of the three truncations were aggregated in inclusion spontaneously regardless of copper treatment (Fig. 4D), suggesting that this way of subdivision of ATG4B might be inappropriate due to the impaired protein structure or incorrect protein folding.

Collectively, the C54 peptidase domain is critical for the formation of ATG4B aggregation.

### 2.5 Copper induces the formation of ATG4B oligomers *in vitro*

**Fig. 5:**
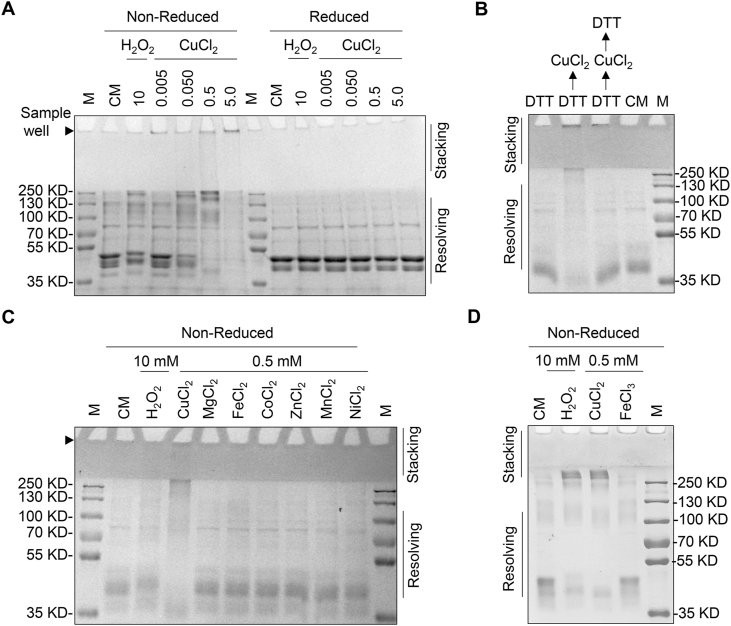
**Copper triggers the formation of ATG4B oligomers *in vitro*.** (**A**) ATG4B protein (4 μg) was treated with designed concentrations of copper ions and H_2_O_2_ (10 mM) at 4 °C for 30 min. The reaction was terminated with 10 mM of NEM to block all cysteine residues and then CBB assay was performed under non-reduced and reduced conditions. (**B**) ATG4B protein (4 μg) were subjected to following treatment respectively: 0.2 mM of DTT at 4 °C for 15 min (lane 1); pretreated with 0.2 mM of DTT followed by 1 mM of H_2_O_2_ for 30 min (lane 2); 2 mM of DTT was added to the mixture as detailed in lane 2 (lane 3); no treatment (lane 4). (**C-D**) ATG4B protein (4 μg) was treated with H_2_O_2_ (10 mM), 0.5 mM of copper ions and indicated metal ions at 4 °C for 30 min. The reaction was terminated with 10 mM of NEM to block all cysteine residues and then CBB assay was performed under non-reduced condition.

We next wondered whether copper can directly act on ATG4B and abrupt its molecular structure followed by the production of ATG4B aggregation. We purified recombinant ATG4B protein without His-Tag to avoid the interference between copper and His-Tag. Then, we directly incubated ATG4B protein with different doses of copper *in vitro* and observed that in non-reduced SDS-PAGE ATG4B proteins distributed in higher molecular weight including oligomers (∼250 kDa) and insoluble stuff (estimated >300 kDa which was unable to enter the stacking gels) (Fig. 5A) Interestingly, the oligomers formed in response to copper disappeared in reduced SDS-PAGE (Fig. 5A). Similarly, we alternatively incubated copper and reducing agent dithiothreitol (DTT) with ATG4B and observed the production of ATG4B oligomer was completely reversed by DTT (Fig. 5B). To validate the specificity of copper-mediated formation of ATG4B oligomer, we further dissected whether other metal ions could induce the similar oligomerization of ATG4B. As shown in Fig. 5C–D, only positive control H_2_O_2_ and copper instead of other metal ions could result in ATG4B oligomer formation. Since copper possessed oxidant property per se and ATG4B could form oligomers under oxidant condition, we employed Fe^3+^ which has higher oxidized potential than copper. We found that different from copper, Fe^3+^ failed to induce the formation of ATG4B oligomers at the same dose under non-reduced conditions (Fig. 5D).

These data suggest that copper could specifically induce the formation of ATG4B oligomers in a redox-dependent manner.

### 2.6 Copper-induced ATG4B oligomers require cysteines

**Fig. 6:**
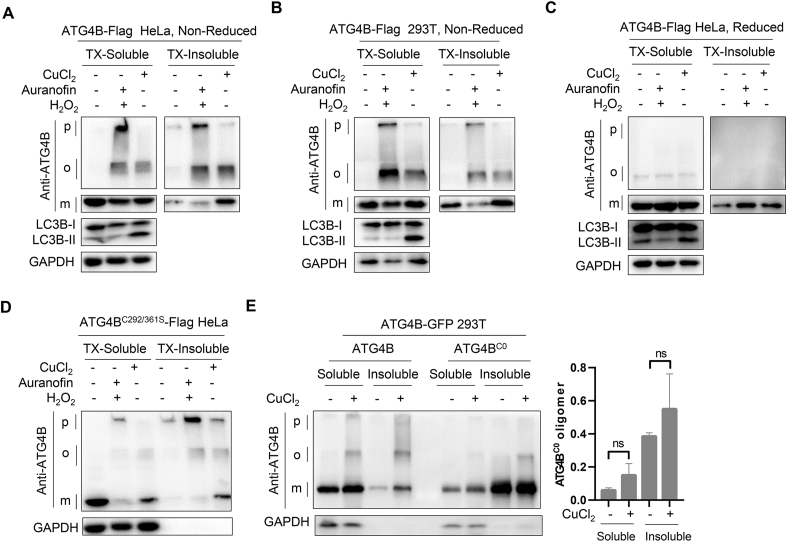
**ATG4B oligomers induced by copper ions are dependent on Cysteines of ATG4B in cells.** (**A-C**) Differential detergent fractionation followed by western blotting under non-reduced or reduced condition was performed on ATG4B-Flag stably expressed HeLa cells or HEK-293T cells pretreated with 5 μM of auranofin and then treated with 1 mM of H_2_O_2_ for 1 h, or treated with 1 mM of copper ions for 6 h (p, pellet; o, oligomer; m, monomer.) (**D**) ATG4B^C292/361S^-Flag stably expressed *ATG4B* KO HeLa cells pretreated with 5 μM of auranofin and then treated with 1 mM of H_2_O_2_ for 1 h, or treated with 1 mM copper ions for 6 h and western blotting was carried out to detect the protein levels under non-reduced condition. Differential detergent fractionation was performed. (**E**) HEK-293T cells stably expressing ATG4B-GFP or ATG4B^C0^-GFP were treated with 1 mM of copper ions for 6 h. Differential detergent fractionation was performed followed by Western blot under non-reduced condition.

We next tested if copper could promote ATG4B oligomer formation in cells. We applied pre-treated auranofin, a suppressor of thioredoxin reductase, combining with H_2_O_2_ as the positive control and observed that consist with *in vitro* assays, ATG4B oligomers increased in both ATG4B-Flag HeLa cells and HEK-293T cells (Fig. 6A–B). Of note, H_2_O_2_ could also cause more insoluble form of ATG4B accumulated in stacking gel, while copper is apt to cause more monomers of ATG4B in TX-Insoluble fraction (Fig. 5, Fig. 6A–B). It shows that the insoluble form of ATG4B is more inclined to form under H_2_O_2_-mediated higher oxidizability and copper could prompt tighter aggregation of ATG4B formation in TX-Insoluble. Similarly, both the H_2_O_2_- and copper-induced ATG4B oligomers were reversed under reduced conditions (Fig. 6C). As we previously reported that the Cys292 and Cys361 are essential for the formation of ATG4B oligomers in response to H_2_O_2_ [13], we wondered whether copper-regulated ATG4B oligomer formation is also dependent on these two sites. We found that ATG4B^C292/361S^ could still form certain oligomers and insoluble form of ATG4B (Fig. 6D), suggesting copper-induced ATG4B oligomer may not depend on Cys292 and Cys361. Given that the formation of protein oligomers is typically mediated by the disulfide bonds between cysteines. We mutated all 14 cysteines to serines of ATG4B as ATG4B^C0^. Although the ATG4B^C0^ is prone to aggregate like other ATG4B truncations in Fig. 4D with unknown reasons, oligomers could not be further induced by copper in ATG4B^C0^ cells (Fig. 6E).

Taken together, these data suggest that, different from H_2_O_2_ which induces ATG4B oligomers require Cys292 and Cys361, copper-induced ATG4B oligomers might involve in other cysteines.

### 2.7 Overexpression of ATG4B partially reverse the formation of MB induced by copper in Wilson disease cell model

**Fig. 7:**
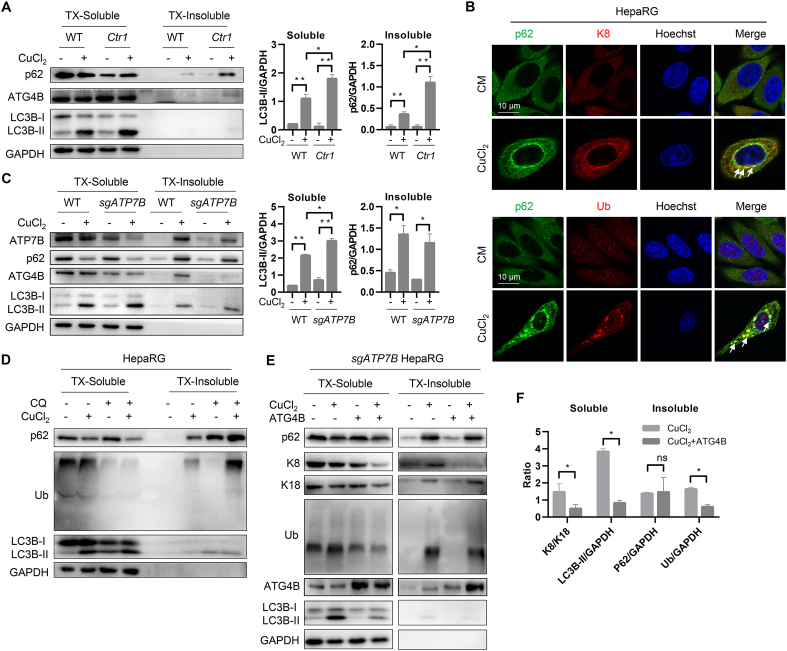
**Copper contributes to the production of MB in Wilson disease through inhibiting autophagy.** (**A**) Differential detergent fractionation was performed on WT HepG2 cells or GFP-Ctr1 HepG2 after treatment with 1 mM of copper ions for 6 h. Western blotting was carried out to detect the protein levels. (**B**) Colocalization analysis of the markers of MB such p62, K8/K18, and Ub before and after treatment of copper ions. The arrows mean colocalization. (**C**) Differential detergent fractionation was performed on WT HepaRG cells or ATP7B knockdown HepRG cells treated with 1 mM of copper ions for 6 h and western blotting was carried out to detect the protein levels. (**D**) Differential detergent fractionation followed by western blotting was performed on HepaRG cells treated with 1 mM of copper ions in the presence or absence of 40 μM of CQ for 6 h. (**E**) Differential detergent fractionation was performed on ATP7B knockdown HepRG cells treated with 1 mM of copper ions for 6 h and western blotting was carried out to detect the protein levels. (**F**) Densitometry analysis of protein bands of (E) was performed. The ratio of K8/K18 and LC3B in soluble fraction, p62 and ubiquitin in insoluble fraction was calculated.

The copper transport receptor 1 (Ctr1) is regarded as a main entry protein for copper intake into eukaryotic cells [42]. We compared the HepG2 cells with or without stably expressing Ctr1 and found that copper-induced more LC3-II in soluble fraction, and ATG4B and p62 aggregates in insoluble fraction when the level of Ctr1 increased (Fig. 7A). In WD, ATP7B was the main protein to cause intracellular copper overload. We observed that p62, ubiquitin, and K18, the main components of MB, were accumulated under copper treatment in L02 cells (Figs. S7A–B), which is corresponded to copper-caused MB formation. Meanwhile, the colocalization ratio between p62 and Ub or K8 are significantly increased after copper treatment in HepaRG cells (Fig. 7B). Next, we generated ATP7B knockdown cells to set up a cell model of WD (Fig. S4C). As expected, ATP7B knockdown with copper treatment further accumulated LC3-II in soluble fraction (Fig. 7C). We then treated HepaRG cells with autophagy inhibitor CQ and found that p62 and ubiquitin-positive aggregates further accumulated due to the impaired autophagy activity (Fig. 7D). It has been reported that autophagy activated by rapamycin could eliminate mouse MB [33]. So, we treated cells with copper plus autophagy activator Torin 1. Intriguingly, Torin 1 failed to eliminate p62 aggregates since autophagy flux has already been disrupted by copper (Fig. S7D). Therefore, we think autophagy suppression promotes MB production, while classic autophagy inducers could hardly eliminate copper-induced MB in our WD cellular model due to the strong inhibition of ATG4B.

It was assumed that copper overload causes MB formation in two different manners. On the one hand, copper-induced oxidative stress contributes to the damage of protein to lead to protein aggregates formation. On the other hand, copper represses the late phase of autophagy degradation via impairing the activity of ATG4B to arrest the clearance of MB. We reasoned whether overexpression of ATG4B could modulate the production of MB either by binding to overloaded copper for detoxification or by reversing defective ATG4B-mediated autophagy suppression. As shown in Fig. 7E–F, the ratio of K8/K18 in the soluble fraction, representing the formation of MB, decreased under ATG4B overexpression compared to copper treatment only. At the same time, lipidated LC3 accumulated by copper decreased significantly due to the increased activity of ATG4B, while the protein level of total ubiquitin in TX-insoluble fraction decreased suggesting the formation of MBs was partially reversed. However, we did not find the level of p62 in insoluble fraction changed before and after overexpression ATG4B, suggesting the degradation of copper-induced p62 aggregation might be not efficient by increased autophagy.

Collectively, these data indicate that suppression of ATG4B by copper inhibits autophagy and promotes MB formation, while overexpression of ATG4B could partially reverse copper overload-induced MBs.

## 3 Materials and methods

### 3.1 Antibodies, reagents, and plasmids

Monoclonal anti-p62/SQSTM1 antibody produced in rabbit (PM045), polyclonal anti-LC3 produced in rabbit (PM036), and monoclonal anti-ATG4B antibody in mouse (M134-3) were from MBL. Polyclonal anti-LC3 produced in rabbit (L7543) and polyclonal anti-ULK1 produced in a rabbit (A7481) were from Sigma. GAPDH monoclonal antibody produced from mouse (60004-1-lg) was purchased from Proteintech. Polyclonal anti-ATP7B produced from rabbit (NB100-360) was purchased from NOVUS. Monoclonal anti-GST antibody produced from mouse (2624) was purchased from Cell Signaling Technology. Monoclonal anti-Ubiquitin (sc-8017) and anti-Cytokeratin 8 (sc-8020) antibodies produced from mouse were from Santa Cruz Biotechnology.

KCl (P5405), NaCl (S9625), CaCl_2_ (499609), MgSO_4_ (M7506), FeSO_4_ (F8633), Fe_2_(SO4)_3_ (307718), ZnCl_2_ (Z0251), CuSO_4_ (451657), CuCl_2_ (222011), MnCl_2_ (M1787), CrCl_3_ (200050), AlCl_3_ (563919), MoO_3_ (NIST423), CoCl_2_ (232696), Na_3_O_4_V (567540), NiSO_4_ (656895) and MG132 (M8699) were purchased from Sigma-Aldrich. Z-VAD-FMK (S7023) were purchased from Selleckchem. CQ (A506569) was purchased from Sangon Biotech. Plasmin (4089) was purchased from Biovision. Kallikrein (A600545) was from Sangon Biotech. Factor Xa (AG00-0102) was from Aglyco. Substrates for serine proteases S2251, S2266, or S2765 were customized by GL Biochem. Substrates for caspases Ac-DEVD-AFC (CASP-048) and Ac-IETD-pNA (CASP-090) were from Chinese Peptide Company. Ac-VDQQD-pNA (P9705), Ac-LEHD-pNA (P9728) was from Beyotime.

### 3.2 Cell culture

HEK-293T (ATCC, CRL-1573) and HeLa cells (ATCC, CCL-2) were grown in Dulbecco’s modified Eagle’s medium (Gibco, 12800082) containing 1% penicillin-streptomycin (Gibco, 15140163) and 10% fetal bovine serum (Gibco, 10270). L02 and HepaRG cells were cultured in RPMI 1640 complete medium (Gibco, 61870036) containing 1% antibiotics and 10% fetal bovine serum. Cells were transfected with Lipofectamine 2000 transfection reagent (Invitrogen, 11668030) according to the manufacturer’s instructions. All cells were cultured at 5% (v/v) CO_2_ at 37 °C in a CO_2_ incubator.

### 3.3 Protein expression and purification

DNA fragments encoding His-tagged ATG4A, ATG4B and FRET-GATE-16 were cloned and inserted into pET-28a (+) as previously described [5]. The above His-tagged proteins were expressed and purified in the same manner. Briefly, pET-28a (+) backboned plasmids were transformed into BL21 (DE3) *E. coli* (Weidi Biotechnology, EC1002). Cells were grown at 37 °C to an OD of 0.6 followed by induction with 0.5 mM of IPTG (Sigma, I6758) at 16 °C. Cells were collected by centrifugation and resuspended in 5 mM imidazole buffer (20 mM Na_3_PO_4_·12H_2_O, 500 mM NaCl, 5 mM imidazole, pH 8.0). Then, cells were lysed by an ultrasonic apparatus and cleared by centrifugation. The supernatant was incubated with Ni-NTA agarose beads (QIAGEN, 30210) for 2 h at 4 °C. Beads were washed and collected with imidazole with different concentrations to wash out unneeded proteins. Proteins were collected and stored at −80 °C in 20% glycerol after desalting.

### 3.4 Preparation of p62 knockout cell lines

To knock out p62 in L02 cells, 2 sgRNA guides containing the targeted sequence were synthesized. *Sgp62* #1: CACCGTATGGCGTCGCTCACCGTGA; *Sgp62* #2: AAACCAGG CTCGGGGCTG CAGCAGC. LentiCRISPR v2 empty vector plasmid (gifts from Junjian Wang, Sun Yat-sen University) was digested by BsmbI-v2 (NEB, R0739S). Guides were phosphorylated using T4 PNK (NEB, M0201S) and then annealed using cooling from 95 °C to room temperature. Finally, guides were ligated into BsmbI-digested LentiCRISPR v2 plasmid using QuickLigase (NEB, M2200S). HEK-293T cells were transfected with lentiCRISPR v2-sgRNA constructs using Lipo2000 according to the manufacturer’s protocol. After 48 h, cells were treated with 2 μg/ml of puromycin (Clontech, 631306). Knockout cells were generated and validated after initial screening.

### 3.5 Immunoblot and immunostaining analysis

Cells were harvested in ice-cold RIPA lysis buffer (Beyotime Biotechnology, P0013B) containing protease inhibitor cocktail (Roche, 04906837001). The proteins in the cell lysates were subjected to SDS-PAGE using a 12% (w/v) gel. The separated proteins were transferred to a PVDF membrane (Invitrogen, 88518). The transferred membrane was blocked with 5% (w/v) skim milk dissolved in TBST buffer (20 mM Tris-HCl, 137 mM NaCl, and 0.1% Tween 20). The membrane was washed three times with TBST buffer and then incubated with primary antibody (1:1000–2000) overnight at 4 °C. The membrane was subsequently visualized by image analyzer (Tanon, 5200) followed by incubation with the secondary antibody.

Cells were cultured on glass-bottom culture dishes (Nest Scientific, 801002). After treatment, cells were fixed with 4% paraformaldehyde for 20 min, and subsequently permeabilized with 0.01% Triton X-100 for 30 min at room temperature. Cells were blocked in fetal goat serum (Boster Biological Technology, AR1009) after washing with PBS for 3 times. Then cells were incubated with primary antibodies (1:100–150) prepared with fetal goat serum overnight at 4 °C. Cells were washed with PBS for three times and incubated with fluorescence-labeled secondary antibody Alexa Fluor 488 or DyLight 594 secondary antibodies (ThermoFisher Scientific, 710369, 35560). Confocal images were observed using a microscope (Olympus, FV3000) equipped with a 100 × oil objective.

### 3.6 Measurement of the inhibition of compounds to ATG4B activity using FRET assay

Purified FRET-GATE-16 and ATG4B were prepared as described previously [7]. Briefly, ATG4B (0.5 μg/ml) and compounds at different concentrations were mixed in 384-well plate (Corning, 3575) and incubated in PBS buffer at 37 °C for 30 min, then FRET-GATE-16 (50 μg/ml) was added into the system with a total volume of 50 μl. The cleavage of the FRET-GATE-16 was measured using a fluorescence spectrometer (Molecular Devices, FlexStation 3) at 37 °C for 30 min. The excitation wavelength was 434 nm and emission was measured at 477 nm (CFP) and 527 nm (YFP or FRET). For detecting the ATG4B activity in cells, cells were collected and dissolved in lysis buffer without protease inhibitor (150 mM NaCl, 1 mM EDTA, 0.1% Triton, 50 mM Tris-HCl, pH 8.0) to avoid the disturbance of ATG4B activity.

### 3.7 Protease activity measurement

For serine protease activity, CuCl_2_ (1 μM) was mixed with 0.1 mg/ml of plasmin, 0.05 mg/ml of kallikrein, or 0.01 U/ml of factor Xa at 37 °C for 30 min, then 0.5 mM of substrates S2251, S2266, or S2765 were added to the above mixture separately. After incubation at 37 °C for another 30 min. The colorimetric change of enzymatic products was recorded by spectrophotometer at 405 nm. For cysteine protease activity, HeLa cells were treated with staurosporine (1 μM) for 5 h to induce cell apoptosis. Then, cell lysates (5 μg for caspase 3) were incubated with/without CuCl_2_ (1 μM) in Tris-buffer in 384-well black plate at 37 °C for 30 min, substrates Ac-DEVE-AFC (25 μM) was then added to a total volume of 50 μl. The cysteine proteases activity was measured at 37 °C for 1 h using a spectrophotometer with excitation of 400 nm and emission of 505 nm for the fluorescence substrate AFC. Cell lysates (20 μg for caspase 2/8/9) were incubated with/without CuCl_2_ (1 μM) in Tris-buffer in 96 well plate at 37 °C for 30 min, substrates Ac-VDQQD-pNA (0.2 mM) for caspase-2, Ac-IETD-pNA (0.2 mM) for caspase 8 or Ac-LEHD-pNA (0.2 mM) for caspase 9 were then added to a total volume of 200 μl. The reaction mixtures were incubated at 37 °C for 3 h, followed by absorbance detection at 405 nm.

### 3.8 Protease-protection assay

The protease-protection assay was performed as previously described [36]. Cells were treated with CuCl_2_ for 24 h prior to collection by trypsinization (Sango biotech, A610629-0050) at 37 °C. Cells were next centrifuged and resuspended in 0.7 ml of ice-cold homogenization buffer (10 mM HEPES-KOH, 0.07 M sucrose, 0.22 M D-mannitol, 1 mM EDTA, 1 mM DTT, pH 7.5). All following steps were performed on ice or at 4 °C. The proteins were extracted by rupturing the cells using 27G needles with a 1 ml syringe for 10 times. Then, cells were centrifuged twice at 300×*g* to discard the nuclear pellet. Each sample was divided into 2 tubes for subsequent permeabilization and counterpart non-permeabilization with 5% Triton X-100 in homogenization buffer to a working concentration of 0.5%. Each 2 samples above were divided into 3 parts, one was treated with 1 mg/ml of trypsin to a final concentration of 100 μg/ml at 37 °C for 30 min. The rest two parts were added with an equivalent volume of homogenization buffer at 37 °C or ice for 30 min. All samples were prepared for further Western blot analysis.

### 3.9 Long-lived protein degradation assay

Long-lived protein degradation assay was performed in accordance with the protocol reported previously [43]. Briefly, HeLa cells were cultured overnight and then incubated with 2 mL of l-methionine-free DMEM (l-methionine-, L-cystatin-, pyruvate-, and glutamine-free DMEM (Thermo Fisher Scientific, 21013024) supplemented with 4 mM of glutamine (BBI Life Sciences, GB0224), 0.2 mM of l-Cystine (Solarbio, C7480), 1 mM of sodium pyruvate (Macklin, S817535) with 10% dialyzed FBS (Biological Industries, 2148391) for 30 min at 37 °C. After incubation, the medium was replaced with l-methionine-free DMEM with 10% dialyzed FBS and 50 μM AHA (MCE, HY-140346A). After incubation for 18 h at 37 °C, 5% CO_2_, culture medium was replaced with regular medium with 10 × L-methionine (Aladdin, M101131) and incubated for 2 h. Then 2 mL of regular medium with 10 × L-methionine was added, cells were treated with drugs for 12 h. Cells were collected and fixed by 1 mL of 4% paraformaldehyde for 15 min at RT. Cells were then permeabilized with 0.5 mL of 0.5% Triton X-100 at RT. Then cells were washed and incubated in the click reaction master mix (50 μM TAMRA alkyne [AAT Bioquest, AAT-487], 1 mM TCEP [Calbiochem, 580560], 100 μM TBTA [GLPBIO, GC45003], 1 mM CuSO_4_ [Sangon Biotech, C3008] in PBS) in the dark for 2 h. After click reaction, cells were centrifuged at 1500 rpm at 4 °C for 10 min to remove the reaction buffer. Finally, cells were suspended in 500 μL of PBS and analyzed by flow cytometry.

### 3.10 Differential detergent extraction

Cells were harvested in ice-cold RIPA lysis buffer (Beyotime Biotechnology, P0013B) containing protease inhibitor cocktail (Roche, 04906837001). Soluble proteins are collected from cell lysates as Triton X-100 soluble fraction after centrifuge (14, 000 rpm) for 10 min. The remaining pellets were washed with ice-cold PBS containing protease inhibitors for three times. The pellets were followed by extracted with SDS lysis buffer (Beyotime Biotechnology, P0013G) containing protease inhibitors extraction as Triton X 100 insoluble fraction. Soluble and insoluble fractions of proteins were separated by Western blot.

### 3.11 Quantification and statistical analysis

For statistical of all quantifications of western blots. Student’s t tests were performed for statistical analysis from at least three independent experiments with Graphpad Prism 8.0 software. Error bars, mean ± SEM. *P < 0.05, **P < 0.01, ***P < 0.001.

## 4 Discussion

ATG4B plays critical roles in autophagy process and could be regulated by various factors, including miRNA, PTMs, protein-protein interactions, and chemical molecules [44]. Both the abundance and insufficiency of ATG4B, such as decreased protein level and restrained enzymatic activity, resulted in suppression of autophagy due to the sequestration of LC3-I and deficient delipidation of LC3-II respectively [38]. In this study, we firstly identified transition metal copper instead of other metal ions that could effectively inhibit the enzymatic activity of ATG4B *in vitro* and in cells. Copper could irreversibly bind to ATG4B. We further demonstrated that autophagy was inhibited with copper treatment due to the inhibition of ATG4B. However, this finding is inconsistent with a recently discovered role of copper in that autophagy is activated in WD [34], which reported that activated autophagy with increased LC3-II and more autophagic structures protected cells from copper-induced cell death. Meanwhile, another published work found that copper is sufficient for autophagy flux in an ULK1/2-dependent manner [35]. In contrast to our findings, they both suggest that copper activates autophagy. Indeed, mTOR, TFEB, and ULK1/2 are more like upstream signals for autophagy initiation rather than late-stage autophagy degradation. In our study, we found early phase of autophagy is activated as well with increased ULK1 positive structures. However, more non-degraded LC3-II and unclosed autophagosomes accumulated under copper treatment. More importantly, copper strongly suppressed LLPD, a classic standard for autophagy function. So we thought that the downstream degradation of autophagy, which they may not detect, is dampened since the strong inhibition of ATG4B in response to copper.

In WD, dysfunction of ATP7B leads to a pathological copper accumulation, especially in the liver and brain, and the formation of MB. Recently, copper chelation therapy is becoming more and more common in the field of copper-overload disease such as WD. Considering the wide range of coordination numbers and geometries of copper, we wondered whether copper induced autophagy inhibition could be reversed by copper chelators. As expected, copper chelator clioquinol reversed the effects of copper on autophagy. Indeed, under basal level (complete medium), the BSA/FBS in the medium chelates a certain amount of copper, so that only high concentrations of copper (＞500 μM) could induce LC3-II in HeLa cells. Previous studies have demonstrated that autophagy activation could eliminate mouse MB [33]. However, the detailed mechanism about how autophagy obliterates MB and whether autophagy plays a role in MB elimination in WD is not clear. In our cell models, copper could negatively regulate autophagy. In addition, we observed that p62 could form insoluble aggregates under copper treatment, which is consistent with previous studies that inactivation of autophagy leads to the formation of cytoplasmic p62 positive protein inclusions [39]. Interestingly, ATG4B could also form protein aggregates in response to copper, suggesting that copper not only inhibits the enzymatic activity but also modify the state of aggregation of ATG4B. We further found copper-regulated ATG4B aggregates formation could not be eliminated by p62 deficiency and ROS scavenger. These suggested that copper might directly regulate ATG4B which is independent of copper-induced oxidative stress. Despite excessive copper accumulation-mediated oxidative stress is an important toxicity mechanism of WD. Soluble ATG4B oligomers formed both *in vitro* and in cells in response to copper tightly relied on cysteines. Different from the H_2_O_2_-induced oligomerization of ATG4B which relies on Cys292 and Cys361, copper-induced oligomers might depend on other more cysteines, but detailed residues of ATG4B as the targets of copper are still unclear. So copper was able to disrupt the protein structure of ATG4B directly to form aggregates and oligomers, and the protease activity, thus inhibiting autophagy. Consequently, impaired autophagy in turn inhibits the clearance of protein aggregates. Since studies have suggested that autophagy inhibitors may prove effective in countering tumor resistance to chemotherapies and suppressing tumor growth, which may prompt the copper-related anti-autophagy compounds exploitation in oncotherapy.

P62 and other protein aggregates are usually thought as pathological characteristics of MB in various diseases [39]. In addition, activated autophagy could assist to eliminate MB in a proteasome inhibitor-mediated model rather than copper treatment model [33]. We then wondered whether deficient autophagy caused by ATG4B inhibition could be recovered to clear out MB in WD cell models when ATG4B is overexpressed. Intriguingly, autophagy inducer Torin 1 failed to activate copper-induced autophagy inhibition due to the impaired deconjugation function of endogenous ATG4B. While overexpression of ATG4B could partially attenuate such autophagy dysfunction and MB formation. Thus, rescuing the late phase of blocked autophagy by enhancing ATG4B activity instead of just activating the early signals of autophagy might be a novel strategy to detoxify MB in WD due to copper overload.

Taken together, we discovered copper not only inhibits the activity of ATG4B directly, but also promotes the oligomerization and aggregation state of ATG4B. Targeting ATG4B-regulated autophagy and enhancement of the activity or protein levels of ATG4B might be a promising way to relieve MB formation in WD.

## Declaration of competing interest

The authors declare that they have no known competing financial interests or personal relationships that could have appeared to influence the work reported in this paper.

## Declaration of competing interest

The authors declare that they have no conflicts of interest.
