# Supplementary material for: Suppression of ATG4B by copper inhibits autophagy and involves in Mallory body formation
Source: Redox Biol. 2022 Mar 24;52:102284. doi: 10.1016/j.redox.2022.102284 (PMC8965161; doi:10.1016/j.redox.2022.102284)

**Supplementary figures**

**Figure S1. Copper inhibits both ATG4B and ATG4A.** (**A-B**) ATG4A (0.5 µg/ml) was incubated with different metal ions (10 µM) respectively for 30 min in a volume of 50 µl at 37℃ followed by adding FRET-GATE-16 (50 µg/ml). The cleavage ratio was calculated (A) and the protein samples were subjected to CBB (B). (**C**) The IC_50_ of CuCl_2_ and CuSO_4_ to ATG4A from fitted curve was tested by FRET. (**D**) Analysis of the protease activity of cysteine proteases caspase-2, -8, -9 and serine proteases plasmin, kallikrein, factor Xa before and after 1 μM of copper treatment. (**E-G**) Surface plasmon resonance was carried out to assess the binding affinity or binding patterns of copper to recombinant proteins ATG4B (E), His-ATG4B (F), and His-ATG4B^C74S^ (G).


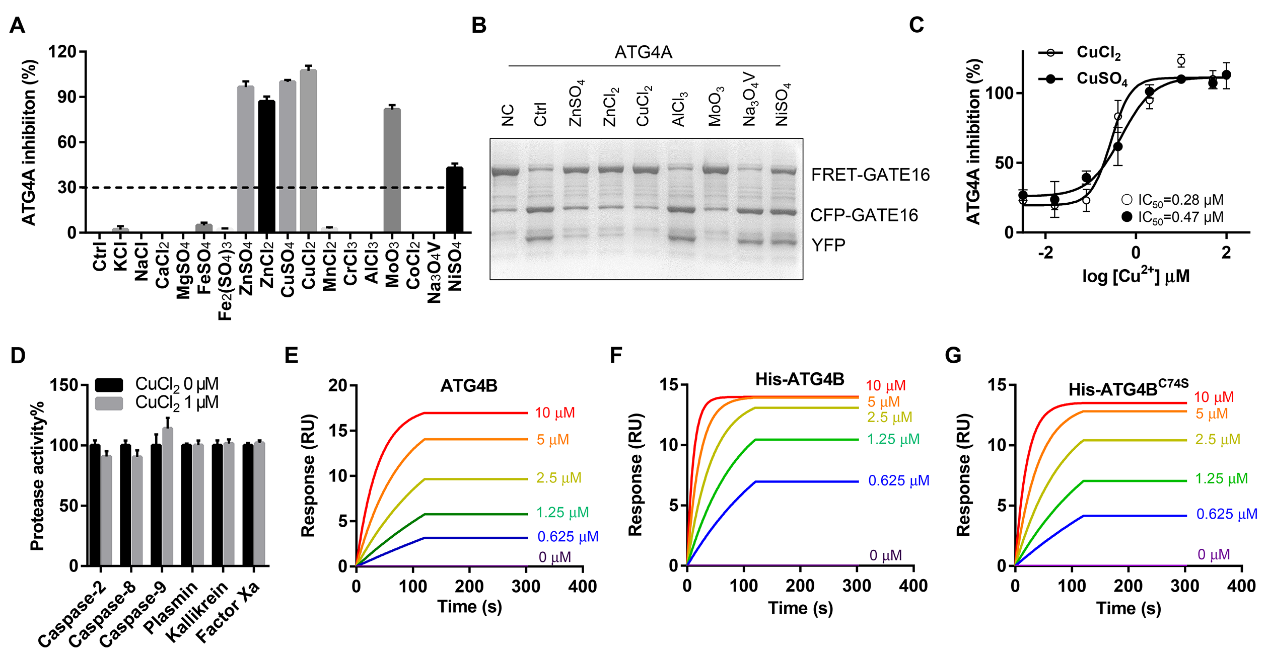


**Figure S2. Copper represses autophagy flux via inhibiting ATG4B.** (**A**) Protease protection assay of homogenates from *ATG4B*KO HeLa cells treated with copper ions (1 mM) for 6 h. (**B**) Diagram of the dual roles of ATG4B in the cleavage of pro-LC3 to LC3-I and LC3-II to LC3-I. PLD and ATG4B cleave LC3-II at different sites. Overexpression of ATG4B has the ability to interact LC3-I as well. (**C**) Western blotting of lysates from WT HeLa cells and *ATG16L1* KO HeLa cells treated with copper ions (1 mM) for 6 h. (**D**) HeLa cells were treated with 4 µM of Torin 1 or 1 mM of copper ions for 6 h. Immunostaining of ULK1 puncta was detected. (**E**) Western blotting of lysates from L02 cells treated with copper ions (0.25 mM) for 6 h in the presence or absence of 20 µM of CQ. LC3-II/GAPDH was calculated based on the bands density.


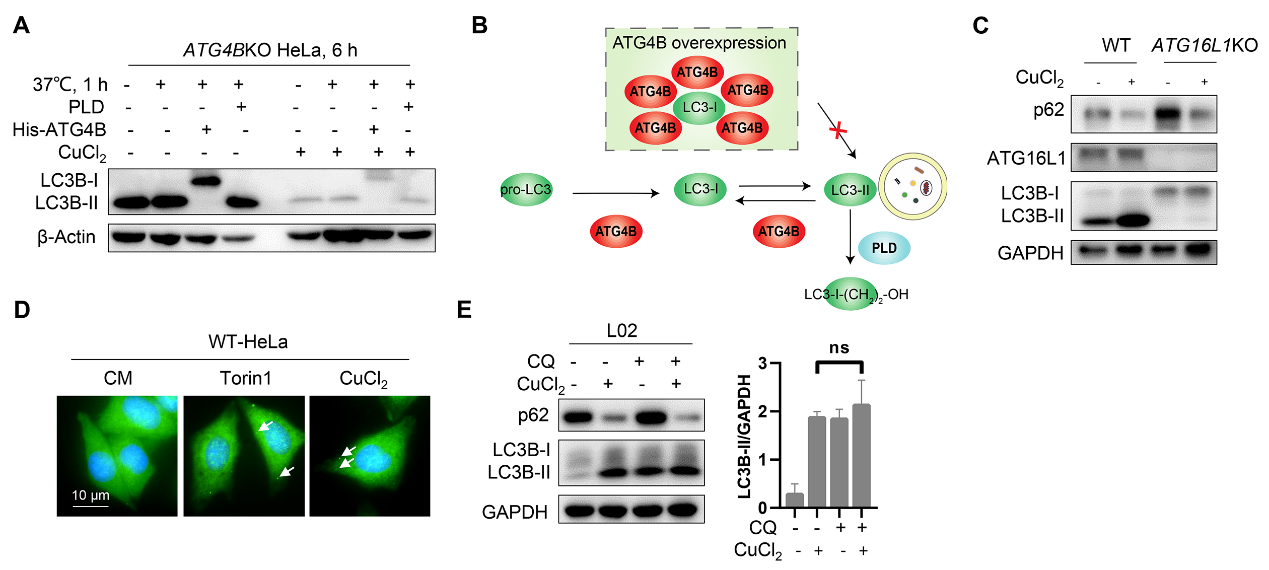


**Figure S3. Copper induces insoluble protein aggregates.** (**A**) Western blotting of cell lysates from L02 cells treated with 0.25 mM of copper ions with or without 10 µM of MG132 for 6 h. (**B**) Differential detergent fractionation was performed on HeLa cells treated with 1 mM of copper ions for given time and western blotting was carried out to detect the protein levels. (**C-D**) Differential detergent fractionation was performed on L02 and HepaRG cells treated with designed concentrations of copper ions for 6 h and western blotting was carried out to detect the protein levels.


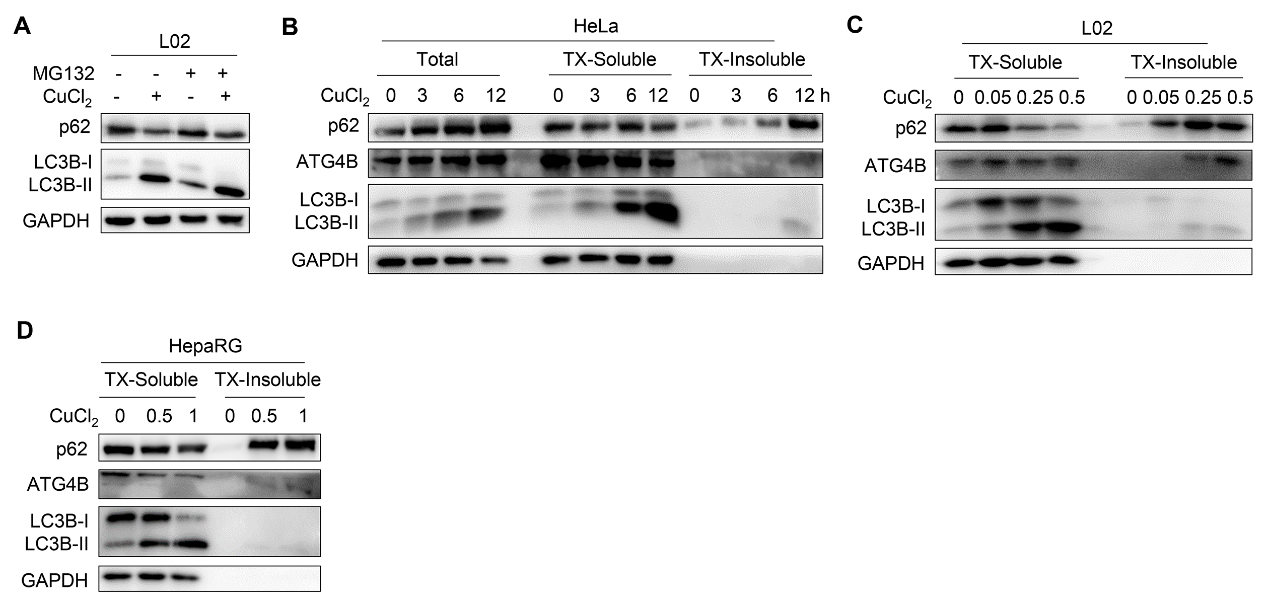


**Figure S4. Copper contributes to the production of MB in Wilson disease cell model.** (**A-B**) Differential detergent fractionation was performed on L02 cells treated with 1 mM copper ions for 6 h and western blotting was carried out to detect the protein levels. (**C**) CRISPR/Cas9 was performed to generate ATP7B knockdown HepaRG cells. (**D**) Differential detergent fractionation followed by western blotting was performed on HepaRG cells treated with 1 mM of copper ions in the presence or absence of Torin1 for 6 h.


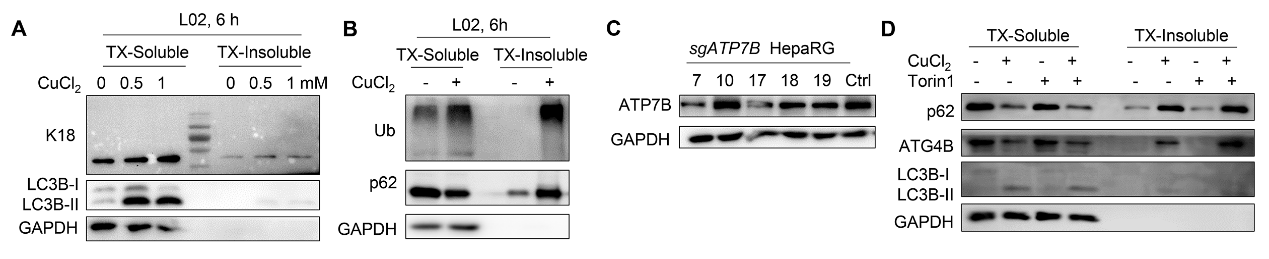

Supplement: Multimedia component 1 [file mmc1.zip › Supplementary data.docx]
