# Supplementary figures and images for: Suppression of ATG4B by copper inhibits autophagy and involves in Mallory body formation
Source: Redox Biol. 2022 Mar 24;52:102284. doi: 10.1016/j.redox.2022.102284 (PMC8965161; doi:10.1016/j.redox.2022.102284)

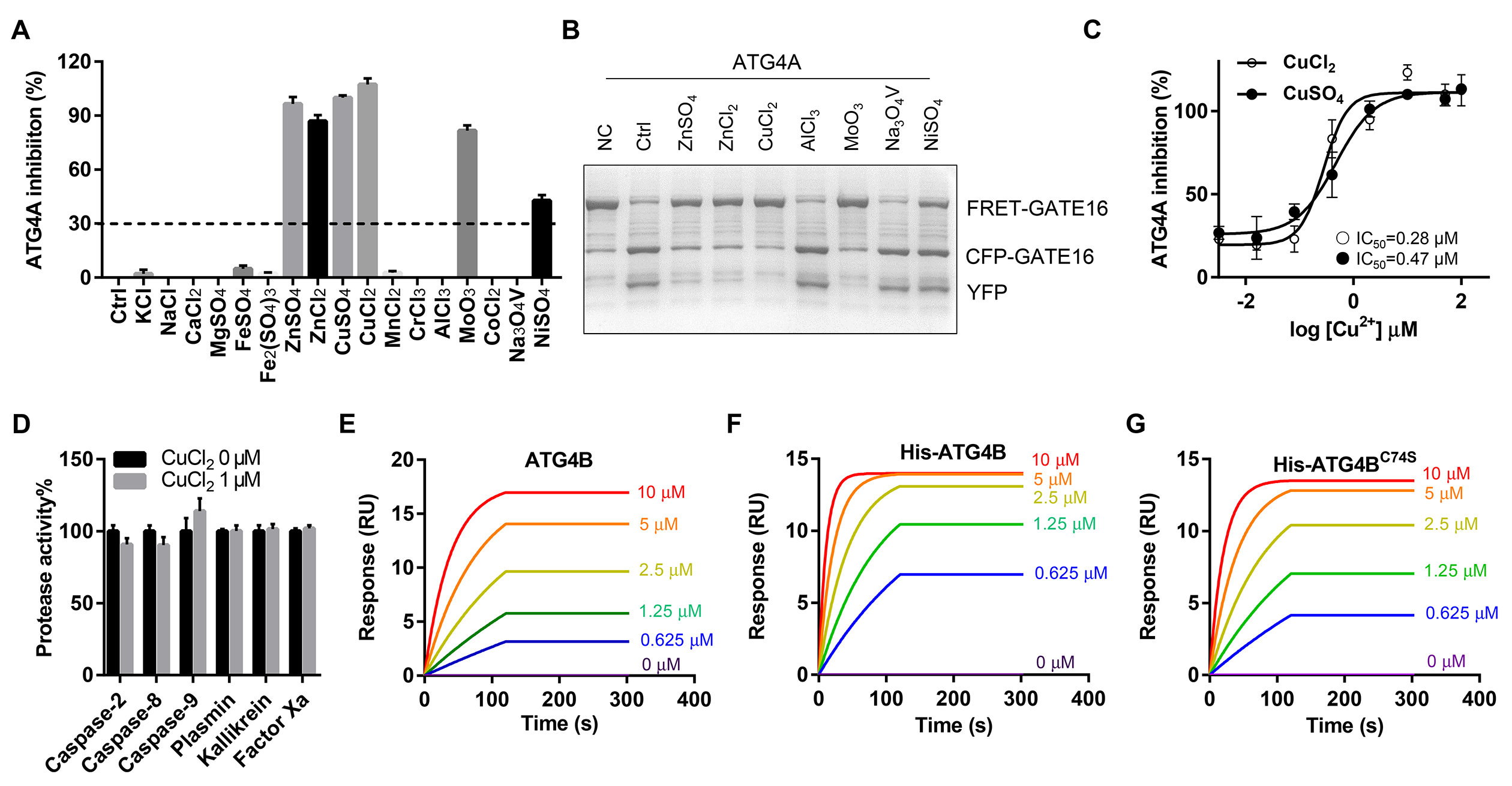

Supplement: Multimedia component 1 [file mmc1.zip › fig.S1.tif]

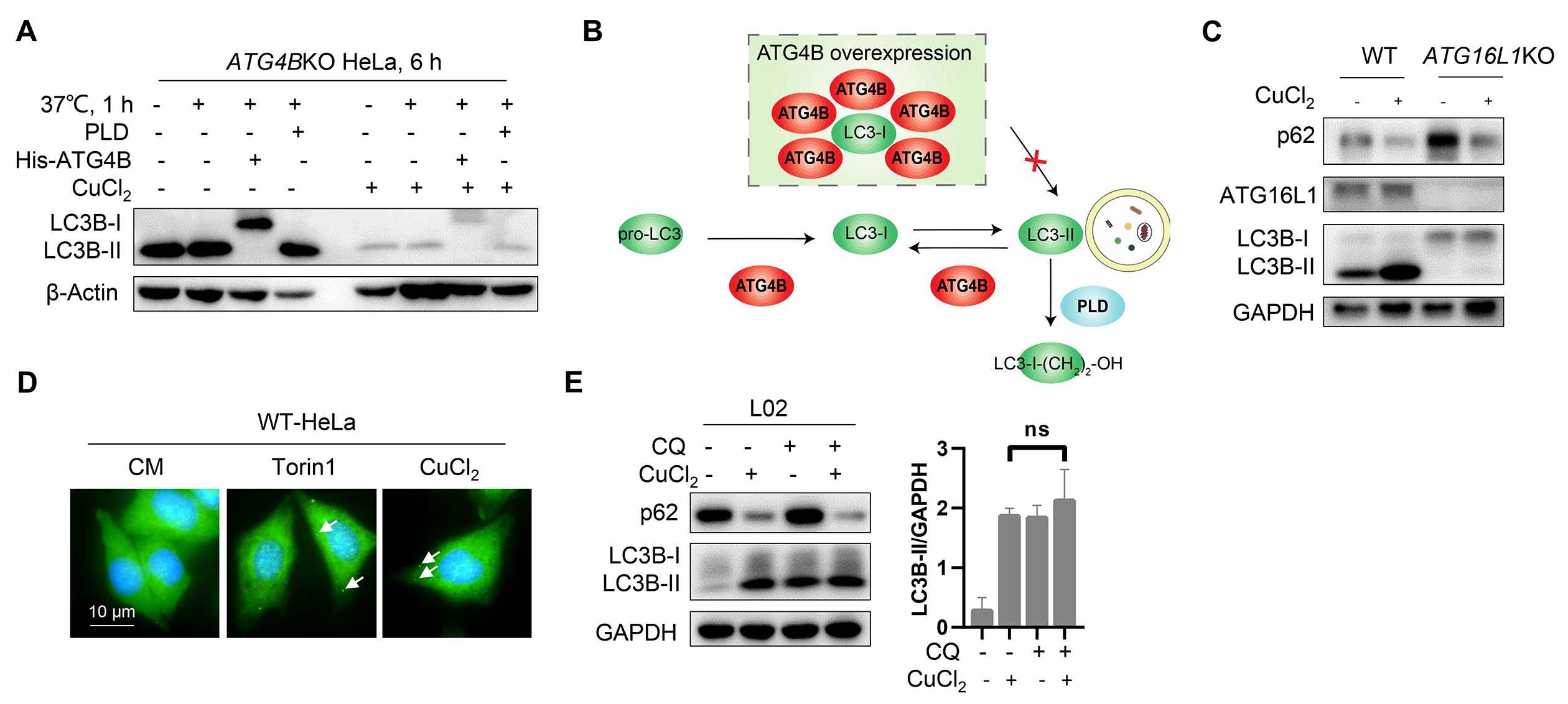

Supplement: Multimedia component 1 [file mmc1.zip › fig.S2.tif]

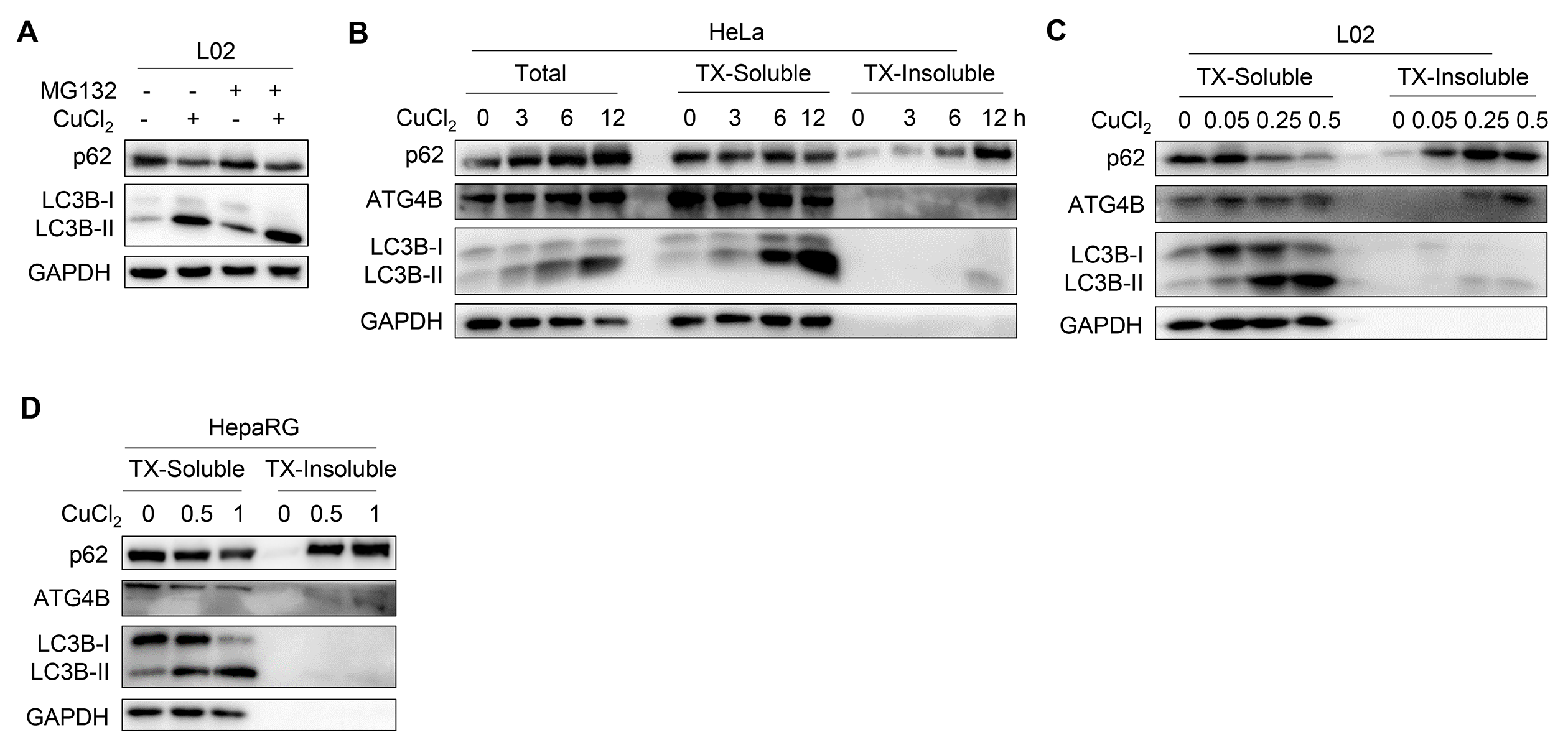

Supplement: Multimedia component 1 [file mmc1.zip › fig.S3.tif]

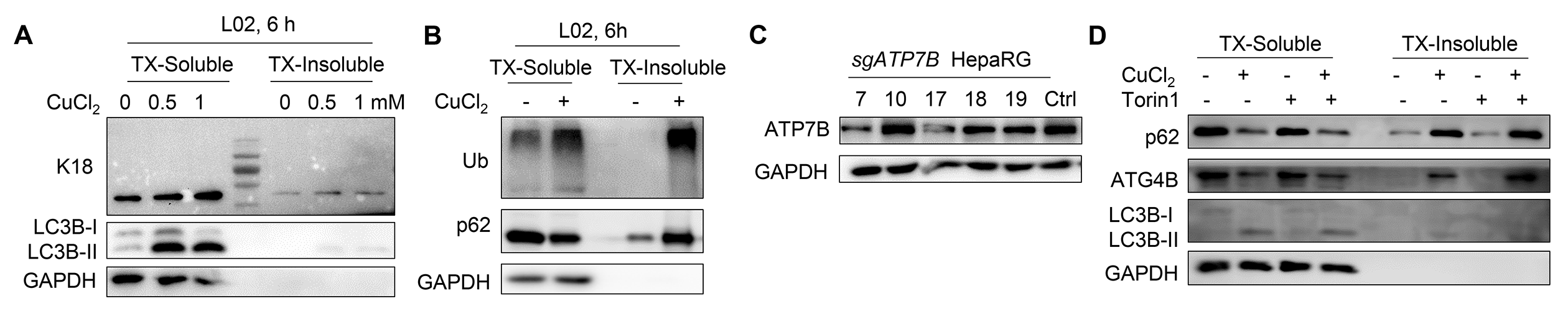

Supplement: Multimedia component 1 [file mmc1.zip › fig.S4.tif]
